# Identification and Characterisation of Lactic Acid Bacteria from “Torta del Casar”, a Semi-Soft Artisanal Cheese

**DOI:** 10.3390/foods15142476

**Published:** 2026-07-13

**Authors:** Micaela Álvarez, María J. Andrade, Francisco Gómez, Alicia Rodríguez

**Affiliations:** 1 Sección Departamental de Nutrición y Ciencia de los Alimentos, Facultad de Veterinaria, Universidad Complutense de Madrid, 28040 Madrid, Spain; malvar54@ucm.es; 2 Higiene y Seguridad Alimentaria, Instituto Universitario de Investigación de Carne y Productos Cárnicos, Facultad de Veterinaria, Universidad de Extremadura, 10003 Cáceres, Spain; mjandrad@unex.es (M.J.A.);; 3 Nutrición y Bromatología, Escuela de Ingenierías Agrarias, Universidad de Extremadura, 06007 Badajoz, Spain; 4 Instituto Universitario de Investigación en Recursos Agrarios (INURA), Universidad de Extremadura, Avd. de la Investigación, 06006 Badajoz, Spain

**Keywords:** 16S rRNA, RAPD-PCR, microbiota, raw milk, starter

## Abstract

“Torta del Casar” is a traditional Spanish cheese made from raw ewe’s milk under Protected Designation of Origin (PDO) regulations. Its unique characteristics result from the use of vegetable rennet and an abundant indigenous microbiota, primarily lactic acid bacteria (LAB). Since PDO rules permit only autochthonous microorganisms, identifying specific LAB strains is a fundamental first step for developing starter and protective cultures. This study aimed to characterise the LAB population of 22 cheeses from different industries and assess batch-to-batch variability. Physicochemical analysis revealed that some batches exceeded PDO pH limits, highlighting the potential need for standardised acidification. Species-level identification was performed via 16S rRNA sequencing, while RAPD-PCR was used for strain-level differentiation. *Lactiplantibacillus plantarum* was the predominant species, followed by *Leuconostoc mesenteroides*. RAPD-PCR differentiated a total of 20 strains across all species, revealing high intra-specific diversity. *Leu. mesenteroides* exhibited higher variability compared to the more homogenous clusters found within *Lpb. plantarum*. Although batches from the same industry showed low strain overlap, specific strains of *Lpb. plantarum* (G5-3, G4-1) and *Leu. mesenteroides* (S2-2, G5-2, L4-1) were shared across different batches or industries. These autochthonous strains are promising candidates for further evaluation as starter cultures, since they could potentially contribute to product standardisation and safety while preserving the traditional sensory profile of “Torta del Casar”.

## 1. Introduction

“Torta del Casar” is a traditional high-quality Spanish cheese produced in six certified cheese dairies in the southwest of Spain in the province of Cáceres under the regulation of the Protected Designation of Origin (PDO) “Torta del Casar” (Casar de Cáceres, Extremadura, Spain) in accordance with Regulation (EC) 1491/2003 of the European Commission [1]. As one of the most emblematic and economically significant dairy products of the Extremadura region, its production is deeply rooted in local heritage. It is a cheese made from raw ewe’s milk belonging to the Merino and Entrefino breeds, which is curdled using a vegetable rennet found in the pistils of the plants *Cynara cardunculus* or *Cynara humilis* [2], without the addition of a starter culture and in the absence of any thermal processing [3,4]. The ripening period of “Torta del Casar” lasts around 60 days, promoting the final characteristics of the product, such as its creamy texture and its unique, strong, slightly bitter flavour and aroma [2]. The development of these characteristics depends on the use of raw milk and vegetable rennet [5,6] as well as the abundant indigenous microbial population [4,7]. Consequently, “Torta del Casar” exhibits a high degree of heterogeneity, as its final properties are strongly influenced by variations in the raw milk and vegetable rennet used on the day it is manufactured.

While some studies have characterised the overall microbiota of “Torta del Casar”, identifying lactic acid bacteria (LAB) as the dominant population followed by Gram-positive catalase-positive cocci (GPCP), yeasts, moulds, and diverse Gram-negative bacteria [2,4], the current literature remains limited to the genus or species level. There is a critical lack of research at the strain level, which is essential, since the physicochemical characteristics and the production of metabolites of interest are strain-dependent [4]. Additionally, undesirable microorganisms such as *Enterobacteriaceae*, coliforms, *Staphylococcus aureus*, or even *Listeria monocytogenes* may be present in the cheese due to the high pH values during the first stages of ripening, its high moisture, and low salt content, raising potential public health risks [2,3,4,8,9]. Consequently, the modern dairy industry increasingly demands product standardisation and safety, which requires strict control of fermentation through the use of autochthonous starters and protective cultures to minimise batch-to-batch variations while preserving traditional sensory attributes.

The indigenous LAB from cheeses are especially well-adapted to the ecological conditions of the processing, controlling the ripening, and inhibiting the growth of undesirable microorganisms [7]. LAB identification is crucial for their use as starters to standardise the production and as protective cultures, as PDO legislation only permits the addition of autochthonous microorganisms to maintain the typical characteristics of the product [10]. A microorganism is considered autochthonous when it is isolated from a typical cheese and is highly adapted to the production area and the traditional technology [11,12].

To address the industry’s need for accurate strain-level selection, phenotypic and biochemical methods are insufficient, as they cannot discriminate between closely related species or differentiate within the same species [13,14,15]. In this scenario, molecular tools are far more consistent, rapid, reliable, and reproducible, and can discriminate even between closely related groups of species that are otherwise indistinguishable based on phenotypic features [15]. For example, the genus formerly known as *Lactobacillus* was reclassified into 23 genera based on new information from advanced molecular techniques [16]. 16S rRNA sequencing is a suitable tool for precise taxonomic identification at the species level [17]. However, to safeguard the diversity of “Torta del Casar” and identify specific robust strains, randomly amplified polymorphic DNA (RAPD) is vital. This approach allows for the screening of a large number of isolates to identify the specific genotypes required by the factories [15]. RAPD is an easy, time-efficient, and affordable method that provides rapid results and gives information about genomic variability below the species level, being the most used technique to differentiate strains [17,18]. For instance, this approach has successfully investigated the genetic diversity of 27 *Leuconostoc* spp. isolates from artisanal Manchego cheeses manufactured at two dairies [19]. The objective of the present study was, therefore, the isolation and identification of autochthonous LAB strains from PDO “Torta del Casar” sourced from different industries, combining 16S rRNA sequencing and RAPD-PCR to perform preliminary strain differentiation and identify potential candidates for the future development of starter and protective cultures.

## 2. Materials and Methods

### 2.1. Cheese Sample Collection

A total of 22 samples (“Torta del Casar” cheeses) were obtained from two different “Torta del Casar” industries under the PDO premises. From the first industry, 5 cheeses from two different batches (S and G) were collected. In the second one, 12 cheeses from the same batch (L) were selected. All the cheeses were collected on the same day, transported to the laboratory, and kept at refrigeration temperature (4 °C) until analysis. Cheese samples were identified by a letter (S, G, or L) and a number. The isolates obtained from each cheese were further coded by adding a second number (e.g., S1-1).

### 2.2. Physicochemical Analyses

In order to evaluate whether the cheeses met the specific characteristics of “Torta del Casar” cheese, a physicochemical assay was performed. The pH was measured on 10 g of each cheese, suspended in 20 mL of distilled water and homogenised manually at room temperature using a digital pH metre electrode (model FC232D; HANNA Instruments S.L.; Eibar, Spain). Water activity (a_w_) was determined by using a Novasina Lab Master water activity metre (Novasina AG., Lachen, Switzerland).

### 2.3. Lactic Acid Bacteria Isolation

To enumerate and isolate LAB, 10 g of each cheese were aseptically homogenised in a sterile stomacher bag containing 40 mL of 0.1% (*w*/*v*) bacteriological peptone (Condalab, Madrid, Spain) for 2 min at 180 rpm. using a Stomacher^®^ 400 Circulator (VWR, Radnor, PA, USA).

Appropriate ten-fold serial dilutions of the samples were prepared with the same diluent, and 100 μL were spread onto the surface of Man-Rogosa and Sharpe Agar plates (MRS; Thermo Fisher Scientific, Waltham, MA, USA). Inoculated plates were anaerobically incubated for 2 days at 30 °C. LAB counts were estimated as Log CFU/g of cheese. It is important to note that this culture-dependent protocol selectively targets the viable and culturable fraction of LAB and does not account for the non-culturable microbial populations present in the cheese matrix.

For isolation and characterisation of LAB, four colonies from MRS agar plates that showed counts between 30 and 300 colonies with different macroscopic morphological features (shape, colour, and size) were randomly selected to represent the dominant culturable population. This sampling strategy focuses on the most prevalent taxa, although it may not capture the full subdominant microbial diversity. Isolates were purified by streaking and stored frozen at −80 °C in Brain Heart Infusion broth (BHI) medium (Condalab, Madrid, Spain) supplemented with 20% (*w*/*v*) glycerol (Thermo Fisher Scientific, Waltham, MA, USA). The selected 88 isolates were routinely cultured on the same medium on which they had been isolated for further assays.

### 2.4. Molecular Identification of Lactic Acid Bacteria

#### 2.4.1. Genomic DNA Extraction

Genomic DNA was extracted using the MasterPure^TM^ DNA Purification Kit (Epicentre, Madison, WI, USA) following the manufacturer’s instructions. Briefly, the protocol involved bacterial cell lysis with proteinase K at 65 °C for 15 min, followed by RNase A treatment at 37 °C for 30 min. Proteins were then removed by precipitation with the MPC reagent and centrifugation. Finally, total nucleic acids were recovered by isopropanol precipitation, washed with 70% ethanol, and resuspended in 35 µL of TE Buffer. The DNA concentration and purity (A_260_/A_280_ ratio) were determined spectrophotometrically using a 2 µL aliquot on the NanoDrop instrument (Thermo Fisher Scientific, Waltham, MA, USA).

#### 2.4.2. 16S rRNA Gene Sequencing for Species-Level Identification

For identification of the isolated strains, two universal primer pairs, Afor (5′-AGAGTTTGATCCTGGCTCAG-3′) and Crev (5′-AAGGAGGTGATCCAGCCGCA-3′) were used for amplifying the 16S rRNA gene [20,21].

PCR amplifications were carried out in a final volume of 50 µL. The final mixture contained 5 µL of 10× reaction Buffer (Takara Bio Inc., Kusatsu, Japan), 2 µL of 10 µM of each primer (Sigma Aldrich, San Luis, MO, USA), 1 µL of 0.2 mM of each deoxynucleoside triphosphate (Roche Diagnostics, Basilea, Switzerland), 3 µL of 50 mM MgCl2 (Thermo Fisher Scientific), 1 U of Taq polymerase (Thermo Fisher Scientific), and 2 µL of DNA template (100 ng/μL). Negative controls containing sterile deionised water instead of the DNA template in each assay were also included. Amplification reactions were performed in an Eppendorf thermal cycler model AG 22331 (Eppendorf, Hamburg, Germany). The PCR conditions were: 1 cycle of 3 min at 94 °C, 30 cycles of 1 min at 92 °C, 1 min at 55 °C, and 1.5 min at 72 °C, and finally 1 cycle of 5 min at 72 °C. After amplification, the presence of amplified products was detected on 2% (*w*/*v*) agarose gel using 1× TAE buffer at 85 V for 1 h. Gels were previously stained with 2 µL Safeview nucleic acid stain RedSafe™ (iNtRON Biotechnology, Seongnam, Republic of Korea) and visualised on a UV transillumination G: Box of Syngene (Cambridge, UK), photographed, and analysed by the integrated camera GeneSnap and the software equipment GeneTools version 08-3d.3 (Syngene). A DNA molecular size marker of 2.1–0.15 kbp (Roche Diagnostics) was used to determine the size of the PCR products.

PCR products were purified and sequenced at the Facility of Bioscience Applied Techniques of SAIUEX (University of Extremadura, Spain) with the same primers used in the amplification steps. Sequencing was performed from both the 5′ and the 3′ ends of each PCR product. The obtained sequences were edited and assembled into a consensus sequence using BioEdit v7.2.5 software. The average length of the assembled sequences was approximately 1400 bp.

To determine the closest known relatives of the obtained 16S rRNA sequences, searches were performed on the GenBank database with the Basic Local Alignment Search Tool (BLAST) programme (http://blast.ncbi.nlm.nih.gov/Blast.cgi). The sequences were analysed separately, and a 99% similarity against type strains was used as the criterion for species identification (Table S1).

#### 2.4.3. RAPD-PCR for Strain-Level Identification

For RAPD-PCR, the microsatellite primer (GACA)_4_ (5′-GACAGACAGACAGACA-3′) was used. Amplification reaction was performed in a total volume of 25 µL containing 12.5 µL of EmeraldAmp^®^ GT PCR Master Mix (TaKaRa Bio Inc., Kusatsu, Japan), 1 µL of 50 µM MgCl_2_ (Thermo Fisher Scientific, Waltham, MA, USA), 2 µL of 10 µM of the primer, 2 µL of DNA template, and 7.5 µL of deionised water. The reaction mixtures were incubated in the thermal cycler using 30 cycles consisting of 1 min at 94 °C, 1 min at 30 °C, and 5 min at 55 °C. To ensure the reproducibility of the technique, 10% of the isolates were analysed in duplicate. The products of PCR were estimated by electrophoresis on 2% (*w*/*v*) agarose gels at 80 V for 1 h, and the products were visualised and photographed as described previously. A DNA molecular marker of 0.5–1.5 kb (NZYDNA Ladder VI. NZYTech. Lda., Lisboa, Portugal) was used to determine the size of the PCR products.

The similarity of banding profiles was analysed using the coefficient of Dice, and the dendrogram was generated with the arithmetic mean UPGMA using NTSYSpc 2.2. software (Exeter Software, Setauket, NY, USA).

## 3. Results and Discussion

Characterisation and identification of the microbial population of traditional cheeses elaborated with raw milk are of utmost interest due to their influence on physico-chemical parameters during the initial stages of ripening and the texture and sensorial properties of the final product [22]. The absence of starter and protective cultures can lead to the growth of undesirable microorganisms that could change the texture and taste of “Torta del Casar” cheese and pose a serious risk to consumers if pathogens develop [4]. Thus, the identification of common strains of LAB from different industries protected by the PDO would facilitate the use of starter and protective cultures without affecting the quality parameters of the product.

### 3.1. Physicochemical Analysis of the Cheeses

The results for a_w_ are shown in Table 1. Lower a_w_ values were observed in cheeses from batch L, which could be attributed to a potentially more advanced degree of ripening [23], whereas batch S presented the highest result. It should be noted that all cheeses were sampled at the end of their maturation period (minimum 60 days), and differences in a_w_ may reflect the inherent variability of the artisanal process. Although significant differences were found between these batches, the values were consistent with previously described data for a_w_ in “Torta del Casar” reported by Ordiales et al. [4]. Similar results have been found for a_w_ in other cheeses elaborated with raw ewe’s milk and vegetable rennet, such as Los Pedroches cheese [23].

According to the results obtained for pH, lower pH values corresponded to batch S, followed by batch G and batch L. According to the PDO legislation, the pH must be in the range of 5.2–5.9. Therefore, only batch S fits perfectly within the appropriate parameters for “Torta del Casar” cheese. The higher pH values observed in batches G and L, especially in the latter, could be attributed to intense proteolysis caused by the vegetable rennet, which releases nitrogenous compounds that increase the pH during ripening [4,24]. Other studies have shown lower pH values within the allowed range for cheese with 60 days of ripening, the minimum time required for its commercialisation [4,24,25]. This deviation from the PDO standards in batches G and L could suggest that standardising the process remains a technological challenge for the sector. Consequently, the autochthonous LAB strains isolated in this study represent preliminary candidates whose future technological characterisation might contribute to a more controlled and consistent acidification process. This could potentially help in maintaining the final product within the legal pH range while enhancing food safety by inhibiting the development of pH-sensitive pathogens.

### 3.2. Microbiological Analyses and Characterisation

All cheeses analysed presented LAB counts between 6 and 8 Log CFU/g, reaching the highest values in batch L. These results are consistent with the LAB counts in MRS medium reported in previous studies for “Torta del Casar” cheese after 60 days of ripening [2,4]. However, it should be noted that the use of MRS agar, which contains glucose as the primary carbon source, is the standard for LAB isolation but might have influenced the selection of specific taxa over those strictly dependent on lactose metabolism. Other cheeses elaborated with raw ewe’s milk and coagulated with vegetable extract, protected by different PDOs such as PDO Serpa, Serra da Estrela, and Queijo de Azeitao, presented similar values for LAB counts, averaging around 7–8 Log CFU/g [26,27,28].

**Figure 1:**
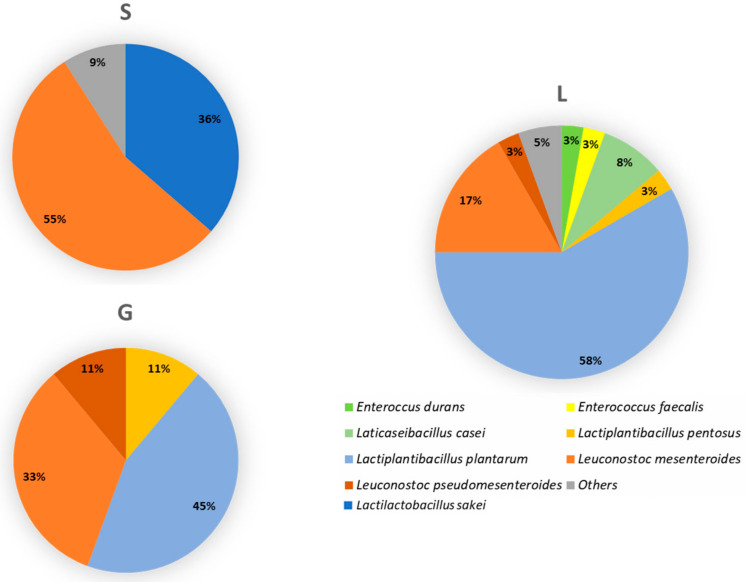
Relative abundance and distribution of lactic acid bacteria species identified by 16S rRNA sequencing in three different batches of “Torta del Casar” cheese. Batches S (*n* = 11 isolates) and G (*n* = 9 isolates) were obtained from Industry 1, while batch L (*n* = 36 isolates) was obtained from Industry 2. “Others” includes species with a prevalence below 3%.

Different microbial populations were found between batches S and G despite belonging to the same industry (Figure 1). In batch S, the predominant species was *Leuconostoc mesenterioides*, followed by *Lactilactobacillus sakei*, while in batch G, the prevalence was the highest for *Lactiplantibacillus plantarum*, followed by *Leu. mesenteroides*. These species have been described as qualified presumption of safety (QPS) biological agents by the European Food Safety Authority (EFSA), so they can be added to food without posing a risk to consumers [29]. Although batch L was obtained from another industry, similar results to batch G were detected, where the main species identified were *Lpb. plantarum* and *Leu. mesenteroides*. The high prevalence of *Lpb. plantarum* in batches from different industries (G and L) points to a frequent occurrence of this species in the specific industrial environments of “Torta del Casar” production, as previously reported for other traditional raw milk cheeses [30,31]. These results differed from other studies about the microbiota of “Torta del Casar”, where the predominant LAB were *Lactococcus lactis* [4,30] and *L. paracasei* [31]. *Lpb. plantarum* has been described as a good candidate for autochthonous starters in PDO Serpa, as it is well adapted to technological conditions and possesses lipolytic and proteolytic activities, which are key for the development of the characteristic sensorial and physicochemical properties of the final product [26]. Furthermore, strains from this species have been reported as biocontrol agents against *L. monocytogenes* in “Torta del Casar” [9] and other types of European cheeses such as Calabrian and Slovak [32,33]. Additionally, different strains of *Lpb. plantarum* isolated from cheeses have been described as promising probiotics [34,35]. *Leu. mesenteroides* was the only species isolated from all three batches in this study. It has been identified in previous studies about “Torta del Casar” and other raw ewe’s milk cheeses elaborated with vegetable coagulants such as Torta de Trujillo [30,31], PDO Queijo de Azeitao [28], and PDO Serpa cheese [36]. This bacterium contributes to the aroma of traditional cheeses because of the production of aromatic compounds, such as 2,3-butanediol and lactate, resulting from the metabolisation of citrate and lactose, and to the texture, probably through the synthesis of dextrans [36]. Additionally, strains of *Leu. mesenteroides* isolated from cheeses have demonstrated their capacity to inhibit *L. monocytogenes*’ growth [37,38]. Another LAB, *Laticaseibacillus casei*, was only found in batch L. This species has been previously identified in “Torta del Casar” and is known for its significant role in the ripening and flavour development of various artisanal cheeses [4].

Regarding the species identified at lower abundance, *E. faecalis* and *Enterococcus durans* present in batch L have been previously isolated from “Torta del Casar” and similar cheeses by other authors [4,30,36]. Although these species are frequently found in raw ewe’s milk cheeses and play an important role in the flavour and texture, their use as protective and starter cultures is controversial due to the potential formation of biogenic amines and the development of antibiotic resistance [4,39]. Therefore, they should be tested for virulence factors and safety characteristics before being recommended as starter cultures [39].

### 3.3. Strain Level Identification

The technological potential of LAB as starter and/or protective cultures on cheeses depends not only on the species, but also on the specific strains used. For example, different strains of *Lpb. plantarum* have shown differences after ripening in PDO Serpa cheese in the pH levels, the formation of free amino acid content, and the volatile profile [40]. Also, the ability of *L. lactis* to produce the bacteriocin nisin against *L. monocytogenes* is also strain-dependent [41]. While previous studies have characterised the microbiota of “Torta del Casar” at the species level, strain-level characterisation remains scarce. Therefore, it is essential to determine whether different industries share the same bacterial strains to apply them as starters without modifying the sensory characteristics of the cheese.

**Figure 2:**
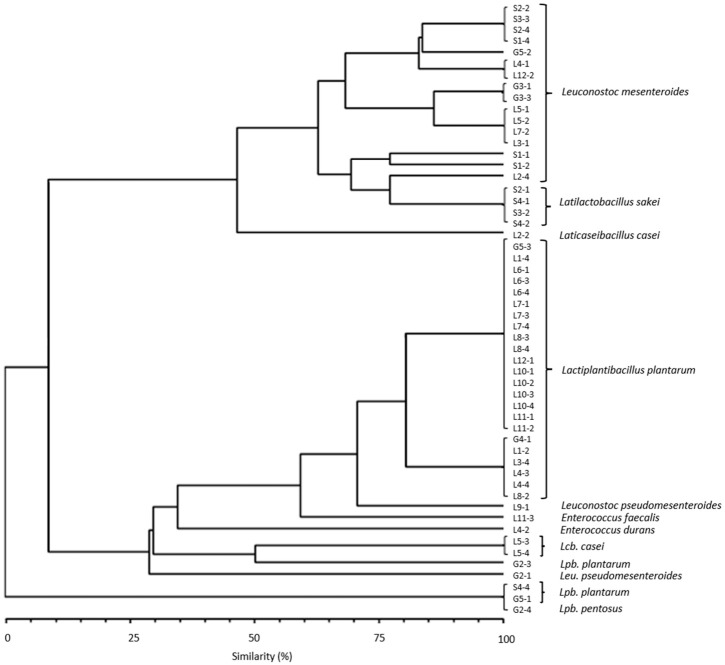
Dendrogram showing the genetic similarity of lactic acid bacteria isolates from “Torta del Casar” cheese (batches S, G, and L). The phylogenetic tree was generated from RAPD-PCR profiles using the (GACA)_4_ primer, based on the Dice similarity coefficient and UPGMA clustering. The vertical line indicates the species-level cut-off. Batches S and G were obtained from Industry 1, while batch L was obtained from Industry 2.

The results of the present study have demonstrated that the LAB population in the studied “Torta del Casar” samples is diverse and batch- and industry-dependent. From the total pool of 88 isolates analysed, RAPD-PCR was able to differentiate 19 distinct patterns that correspond to 20 strains/genotypes of LAB (Figure 2): eight strains of *Leu. mesenteroides*, one *Llb. sakei*, two *Lcb. casei*, four *Lpb. plantarum*, two *Leu. pseudomesenteroides*, one *E. faecalis*, one *E. durans*, and one *Lpb. pentosus*. The low similarity observed between strains of the same species, particularly within *Lpb. plantarum*, justifies the use of RAPD-PCR as a necessary tool to reveal intra-specific biodiversity that 16S rRNA sequencing alone would not have detected.

The dendrogram shows the similarity between strains (Figure 2). Despite batches S and G coming from the same industry, only one strain of *Lpb. plantarum* was shared between them (S4-4 and G5-1). Notably, this specific strain (S4-4/G5-1) formed a separate cluster at the bottom of the dendrogram, showing very low similarity (less than 25%) with the other *Lpb. plantarum* isolates, which highlights the high genetic diversity within this species in the PDO area. On the other hand, since the *Lpb. pentosus* strain G2-4 shared the exact same fingerprinting pattern as the neighbouring *Lpb. plantarum* strains, it is highly probable that this isolate was misidentified by 16S rRNA sequencing due to the high sequence homology within this taxonomic group. When compared across different industries, batches S and L had no strains in common, but in batches G and L, two major strains of *Lpb. plantarum* were detected in both (G5-3 and G4-1). As shown in Figure 2, strain G5-3 was identical to a large group of isolates from batch L (e.g., L1-4, L6-1, L10-1), while strain G4-1 was identical to another cluster from batch L (e.g., L1-2, L3-4). The identification of identical strains (G5-3 and G4-1) in different industries suggests a frequent occurrence and potential adaptation within the sampled facilities, making them preliminary candidates for further technological evaluation (including acidification kinetics and proteolytic activity) as a standardised starter culture.

Similarly, *Leu. mesenteroides* strains exhibited significant genetic consistency across different origins. Specifically, a cluster comprising strains S2-2, G5-2, and L4-1 showed a similarity of over 80% (Figure 2), despite these isolates coming from different batches and industries. The ubiquity of these closely related *Leu. mesenteroides* genotypes suggests they are a relevant part of the microbiota of the studied dairies, pointing to their potential role as components of the indigenous microbiota of “Torta del Casar”, likely well-adapted to the specific ecological niche of raw ewe’s milk and vegetable rennet coagulation. The presence of the same strains in different industries could be due to the milk’s origin, which can come from the same farms grouped under a cooperative regulated by the PDO [30]. However, data from the same industry (batches S and G) suggested that other factors could be involved in the heterogeneous LAB population of “Torta del Casar”, such as the microbiota of *Cynara cardunculus* and interactions between microorganisms during ripening [4,25,30,42].

It is worth noting that only a single batch was analysed from industry L, which represents a limitation of this study, as it precludes a full assessment of intra-industry variability at this specific facility. Additionally, the selection of four colonies per sample focuses on the dominant population of culturable LAB but may not fully reflect subdominant microbial diversity, a factor that should be considered when defining the core microbiota of the cheese. Therefore, future research encompassing multiple successive batches from all six active dairies and a higher number of isolates per sample would be essential to thoroughly validate these industrial dynamics and provide a more comprehensive characterisation of the microbial ecosystem.

These results corroborate the microbiota diversity in raw milk cheeses, despite being made in the same dairy or with milk from the same farm. To standardise production, strains that have been consistently isolated from different batches, such as *Lpb. plantarum* G5-3, G4-1, and S4-4, along with the dominant *Leu. mesenteroides* strains (S2-2, G5-2, and L4-1) could be potential candidates for future research regarding their use as autochthonous starters in “Torta del Casar”.

## 4. Conclusions

This study provides a preliminary characterisation of the LAB population of “Torta del Casar” at the strain level. The results indicate that microbial differences exist not just at the species level but significantly at the strain level, particularly within the dominant species *Lpb. plantarum* and *Leu. mesenteroides*, even within cheeses from the same industry. Although based on a limited number of industries and isolates, these preliminary results underscore the potential technological necessity of implementing autochthonous starter cultures to potentially improve product standardisation and safety. This highlights the utility of strain-specific selection using molecular tools like RAPD-PCR to reveal intra-specific biodiversity that remains hidden with 16S rRNA sequencing alone.

Focusing on the most representative taxa identified, strains such as *Lpb. plantarum* G5-3, G4-1, and S4-4, along with the *Leu. mesenteroides* S2-2, G5-2, and L4-1 represent promising candidates for future technological and safety studies. The potential use of these autochthonous strains could eventually allow for standardising cheese production, ensuring compliance with PDO regulations. However, further comprehensive studies regarding their technological performance (e.g., acidification kinetics, proteolytic and antimicrobial activity), volatile compound production, and safety profiles (e.g., absence of virulence factors and antibiotic resistance) are essential and mandatory before considering their full-scale industrial application as starter and protective cultures.

## Figures and Tables

**Table 1:** Physicochemical characteristics (a_w_, pH) and lactic acid bacteria (LAB) counts of the different batches of “Torta del Casar” cheeses.

Industry	Batch	a_w_	pH	LAB Counts (Log CFU/g)
1	S	0.95 ± 0.00 ^a^	5.67 ± 0.11 ^c^	6.17 ± 0.00 ^c^
1	G	0.93 ± 0.00 ^b^	6.32 ± 0.18 ^b^	7.16 ± 0.00 ^b^
2	L	0.91 ± 0.01 ^c^	6.88 ± 0.29 ^a^	8.03 ± 0.25 ^a^

Statistical differences between batches are indicated with different letters.

## Data Availability

The original contributions presented in the study are included in the article/Supplementary Materials. Further inquiries can be directed to the corresponding author.

## Supplementary Materials

The following supporting information can be downloaded at: https://www.mdpi.com/article/10.3390/foods15142476/s1. Table S1. Genetic identification of lactic acid bacteria strains isolated from “Torta del Casar” cheese (batches S, G, and L) by 16S rRNA gene sequencing.
