# Supplementary material for: Identification and Characterisation of Lactic Acid Bacteria from “Torta del Casar”, a Semi-Soft Artisanal Cheese
Source: Foods. 2026 Jul 13;15(14):2476. doi: 10.3390/foods15142476 (PMC13409492; doi:10.3390/foods15142476)
Supplement: Supplementary file 1 [file foods-15-02476-s001.zip › foods-4396165-supplementary.pdf]

Table S1. Genetic identification of lactic acid bacteria strains isolated from “Torta del Casar” cheese (batches S, G, and L) by 16S rRNA gene sequencing.

Strain	Identification	GeneBank accession number	Similarity (%)
G5-1	*Lactiplantibacillus plantarum*	NR_104573.1	92.43
G2-3	*Lpb. plantarum*	NR_104573.1	97.00
G2-4	*Lactiplantibacillus pentosus*	NR_029133.1	99.60
G3-1	*Leuconostoc mesenteroides*	NR_074957.1	99.49
G4-1	*Lpb. plantarum*	NR_104573.1	90.16
G5-2	Leu. mesenteroides	NR_074957.1	97.29
G5-3	*Lpb. plantarum*	NR_115605.1	95.61
L1-2	*Lpb. plantarum*	NR_104573.1	95.26
L4-1	*Leu. mesenteroides*	NR_074957.1	95.51
L6-3	*Lpb. plantarum*	NR_115605.1	95.11
L9-1	*Leuconostoc pseudomesenteroides*	NR_109004.1	96.86
L11-3	*Enterococcus faecalis*	NR_113901.1	98.47
L1-4	*Lpb. plantarum*	NR_115605.1	99.59
L2-1	*Latilactobacillus sakei*		
L2-2	*Laticaseibacillus casei*	NR_113333.1	99.458
L3-4	*Lpb. plantarum*	NR_104573.1	99.50
L4-2	*Enterococcus durans*		
L4-4	*Lpb. plantarum*	NR_104573.1	98.90
L5-1	*Leu. mesenteroides*	NR_074957.1	98.86
L5-2	*Leu. mesenteroides*	NR_074957.1	100.00
L5-3	*Lcb. casei*	NR_113333.1	99.27
L6-1	*Lpb. plantarum*	NR_115605.1	99.81
L6-4	*Lpb. plantarum*	NR_115605.1	99.66
L7-1	*Lpb. plantarum*	NR_115605.1	98.81
L7-2	*Leu. mesenteroides*	NR_074957.1	99.18
L7-4	*Lpb. plantarum*	NR_115605.1	98.86
L8-3	*Lpb. plantarum*	NR_115605.1	99.01
L8-4	*Lpb. plantarum*	NR_115605.1	99.17
L10-2	*Lpb. plantarum*	NR_115605.1	99.49
L10-3	*Lpb. plantarum*	NR_115605.1	96.05
L10-4	*Lpb. plantarum*	NR_115605.1	98.71
L11-1	*Lpb. plantarum*	NR_115605.1	93.10
L11-2	*Lpb. plantarum*	NR_115605.1	99.31
L12-1	*Lpb. plantarum*	NR_115605.1	99.32
L12-2	*Leu. mesenteroides*	NR_074957.1	99.18
S1-1	*Leu. mesenteroides*	NR_074957.1	94.46
S3-2	*Llb. sakei*	NR_113821.1	95.74
S4-1	*Llb. sakei*	NR_113821.1	97.11
S1-2	*Leu. mesenteroides*	NR_074957.1	99.40
S1-4	*Leu. mesenteroides*	NR_074957.1	98.58

S2-4	*Leu. mesenteroides*	NR_074957.1	98.80
S3-3	*Leu. mesenteroides*	NR_074957.1	99.32
S4-2	*Llb. sakei*	NR_113821.1	98.67
S4-4	*Lpb. plantarum*	NR_104573.1	98.39
